# Same actors, same processes, same outcomes: Global health architecture reform or restoration?

**DOI:** 10.1371/journal.pgph.0006781

**Published:** 2026-07-08

**Authors:** Indira Dewi Kantiana, Filipa Alpeza, Shashika Bandara, Alex R. Cook, Afifah Rahman-Shepherd

**Affiliations:** 1 Saw Swee Hock School of Public Health, National University of Singapore and National University Health System, Singapore, Singapore; 2 Stockholm School of Economics, Stockholm, Sweden; 3 Jeffrey Cheah School of Medicine and Health Sciences, Monash University Malaysia, Kuala Lumpur, Malaysia; PLOS: Public Library of Science, UNITED STATES OF AMERICA

*“Power concedes nothing without a demand. It never has and it never will.” – Frederick Douglass*

On 22 May 2026, the 79^th^ World Health Assembly (WHA) adopted the proposal for a joint, inclusive, transparent and time-bound process, hosted by WHO, to support reforms of the global health architecture (GHA) [1]. The proposal outlines a process to develop options and recommendations for aligning mandates and capacities of GHA actors with essential GHA functions, and enhancing coordination to strengthen collaboration, accountability and coherence [2]. A 25-member Taskforce, comprising 14 Member State representatives and 11 non-Member State representatives, will develop two reports (interim and final) containing a roadmap for implementation for consideration at the 80^th^ WHA in 2027. We question whether this process is designed to genuinely reform the GHA beyond the status quo by unpacking the composition of the Taskforce, as well as the accountability and scope of the joint process.

## Subjects of reform are also designing the reform

The proposal advocates for a GHA that is ‘truly country-led’, yet almost half the Taskforce are not Member States, but representatives from five global health initiatives, up to four UN entities (including WHO), the World Bank, and one regional health organisation. The actors whose mandates, financing, and institutional survival are the subject of reform are the very same actors being tasked with developing options and recommendations for reform. While their buy-in is essential, the process must recognise their disproportionate influence, political capital, and financial leverage in global health [3]. Any meaningful reform to shift the status quo will require some level of sacrifice of power by actors within these institutions [4]. Giving the principals of these institutions a seat at the table, without strategies to manage their influence, risks reproducing inequitable power dynamics between funders, implementers, technical agencies and countries of the global north and global south that have long defined these decision-making spaces. Not even civil society, who typically hold power to account, are included in the Taskforce—after the second round of consultations, the Taskforce was stripped of civil society (and youth) representation, now only engaging them through ‘regular meetings’ [2].

## Accountability is absent from the outset

The proposal provides timelines, deliverables, and activities for four phases of work, but lacks a monitoring, evaluation, and learning framework, as well as an independent oversight mechanism to ensure alignment between the process, its purpose and principles. The proposal acknowledges risks, like ‘uneven’ participation and potential conflicts of interest, yet provides no guardrails to manage them proactively. Instead, consultations with Member States will be held in Geneva, the interim report will be considered by the Boards of the 11 non-Member States representatives, and an advanced copy of the final report will be shared with the Boards of the five global health initiatives before submission to the 80^th^ WHA [5,6]—establishing, again, patterns of privilege in global health decision-making spaces. Who will oversee a process where the actors proposing options for reform are the same actors meant to implement them? Who will assess and manage potential conflicts of interest, and how? Who will ensure meaningful participation and transparent documentation of the process? Without embedding accountability from the outset, the integrity of this process and credibility of its outcomes cannot be assured.

## Limited scope undermines reform ambition

While the Taskforce may provide clarity on GHA functions, map mandates and capacities, and offer options to address overlaps and gaps, the proposal makes it clear the process will propose ‘neither revisions to organisational mandates nor specific mergers or consolidations, which fall within the authority of the relevant governing bodies’ [2]. Yet surely since many of these ‘relevant governing bodies’ are members of the Taskforce themselves, they are in an ideal position to propose such revisions and structural changes to their own organizations. Limitation to the scope, perhaps intended to respect each institution’s governance structure, might instead be indicative of a pre-determined interest to survive. Across the GHA, institutions have a well-documented history of acting in self-preservation, for example, by expanding their mandates beyond their original scope of work [3]. Even institutions that have publicly supported mandate sunsets also make it clear that reforms should retain certain functions with their institution’s mandate [7]. If the scope is not sufficiently ambitious, the options and recommendations for reform will cede to the interests of the institutions being asked to reform, rather than the purpose of reform itself.

## Where do we go from here?

**Fig 1:**
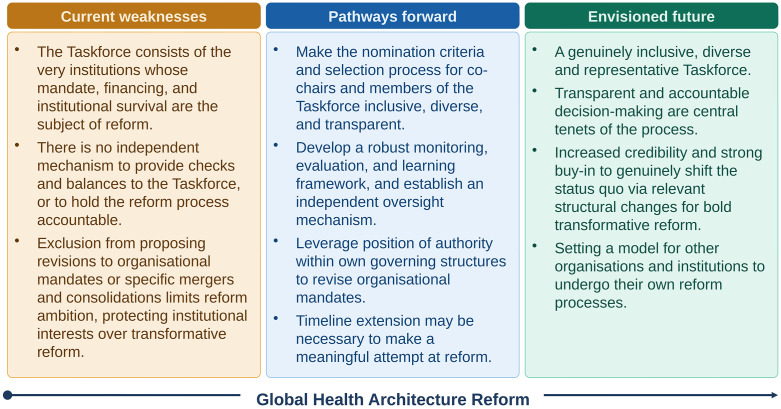
Summary of the current weaknesses, pathways forward, and envisioned future for GHA.

While the WHA decision indicates consensus on the need for a reform process, the issues raised regarding the composition of the Taskforce, as well as the accountability and scope of the joint process, allude to a lack of honest willingness to address existing weaknesesses in the GHA [8]. If the process fails to acknowledge its criticisms early and make critical adjustments before moving forward, weakened credibility will lose the buy-in it needs for success [9,10]. While we recognise that the proposal has already been adopted, limiting the extent to which it can be substantively changed, we urge the Taskforce to make four critical adjustments. First, the WHO Secretariat must make the nomination criteria and selection process for co-chairs and members of the Taskforce inclusive, diverse, and transparent. Criteria should amplify voices of communities, institutions and countries disproportionately affected by inequities in health, and encourage nominations beyond political and diplomatic competence, including when establishing the time-limited working groups [11]. Second, the selected co-chairs and representatives of the Taskforce must hold the reform process to account by developing a robust monitoring, evaluation, and learning framework, and establishing an independent oversight mechanism that can track meaningful participation, promote transparent reporting, and manage conflicts of interest within the Taskforce [12]. Third, the process must not shy away from developing options and recommendations that include revisions to organisational mandates or specific mergers and consolidations. The 11 non-Member State representatives on the Taskforce must reaffirm their commitment to bold reforms of the GHA and leverage their positions of authority within their own institutional governing structures to genuinely re-examine their institutions’ mandates and capacities [13]. Fourth, while a one-year timeline can help to maintain momentum and accelerate action on the reform agenda, the urgency to deliver should not undermine the quality of the process. A meaningful process that takes into account these adjustments may require extending the timeline. The Taskforce could initially consider extending the ‘set-up’ phase (June-July 2026), which is a crucial window for planning and preparations, and subsequent extensions at the interim report stage, giving ample time for consultations and feedback. Without considering these adjustments (see Fig 1), the process is at risk of simply repackaging the same actors, processes, and outcomes as yet another resource-intensive attempt to maintain the status quo [14].
